# UDP-Glycosyltransferases and Albendazole Metabolism in the Juvenile Stages of *Haemonchus contortus*

**DOI:** 10.3389/fphys.2020.594116

**Published:** 2020-11-26

**Authors:** Pavlína Kellerová, Martina Navrátilová, Linh Thuy Nguyen, Diana Dimunová, Lucie Raisová Stuchlíková, Karolína Štěrbová, Lenka Skálová, Petra Matoušková

**Affiliations:** Faculty of Pharmacy, Department of Biochemical Sciences, Charles University, Hradec Králové, Czechia

**Keywords:** UGT, biotransformation, drug resistance, nematode, anthelmintics, UHPLC-MS, gene expression

## Abstract

The nematode *Haemonchus contortus*, a gastrointestinal parasite of ruminants, can severely burden livestock production. Although anthelmintics are the mainstay in the treatment of haemonchosis, their efficacy diminishes due to drug-resistance development in *H. contortus.* An increased anthelmintics inactivation via biotransformation belongs to a significant drug-resistance mechanism in *H. contortus*. UDP-glycosyltransferases (UGTs) participate in the metabolic inactivation of anthelmintics and other xenobiotic substrates through their conjugation with activated sugar, which drives the elimination of the xenobiotics due to enhanced solubility. The UGTs family, in terms of the biotransformation of commonly used anthelmintics, has been well described in adults as a target stage. In contrast, the free-living juvenile stages of *H. contortus* have attracted less attention. The expression of UGTs considerably varies throughout the life cycle of the juvenile nematodes, suggesting their different roles. Furthermore, the constitutive expression in a susceptible strain with two resistant strains shows several resistance-related changes in UGTs expression, and the exposure of juvenile stages of *H. contortus* to albendazole (ABZ) and ABZ-sulfoxide (ABZSO; in sublethal concentrations) leads to the increased expression of several UGTs. The anthelmintic drug ABZ and its primary metabolite ABZSO biotransformation, tested in the juvenile stages, shows significant differences between susceptible and resistant strain. Moreover, higher amounts of glycosidated metabolites of ABZ are formed in the resistant strain. Our results show similarly, as in adults, the UGTs and glycosidations significant for resistance-related differences in ABZ biotransformation and warrant further investigation in their individual functions.

## Introduction

The nematode *Haemonchus contortus* is a problematic and highly successful parasite of small ruminants. One female worm can produce between 5,000–15,000 eggs per day, and if the infected animal is left to roam freely on the pasture, the spread of *H. contortus* can be enormous (Kaplan and Vidyashankar, 2012). *H. contortus* adults cause haemonchosis, with chemotherapy remaining the primary solution for the treatment of infected animals. Despite the availability of several classes of anthelmintics, this parasite has demonstrated a remarkable ability to develop resistance to any drug introduced into the market so far (Besier et al., 2016; Kotze and Prichard, 2016). No doubt, the principal mechanism of benzimidazole anthelmintics resistance is the mutation of their target site, the beta-tubulin 1 (Shalaby, 2013). Several other drug-resistance mechanisms in *H. contortus* have been described, with one of these based on increased deactivation and/or efflux of anthelmintics via the increased expression of drug-metabolizing enzymes. Benzimidazole anthelmintics such as albendazole (ABZ) and flubendazole (FLU) are metabolized by *H. contortus* adults into several inactive derivatives through S-oxidation, reduction, methylation, N-acetylation as well as several types of *N*- and *O*- glycosidation (Stuchlíková et al., 2018). Glycosidation or conjugation of activated sugar to a lipophilic compound, in general, is performed by UDP-glycosyltransferases (UGTs), during the second phase of biotransformation, causing the metabolic inactivation of xenobiotic substrates. Such a conjugation drives the elimination of the compound due to enhanced solubility. The important role of UGTs in resistance to xenobiotics has been described in a variety of organisms, especially in various pests, e.g., weeds for which the resistance represents a serious burden to agriculture, where UGTs are involved in the metabolic deactivation of herbicides to non-phytotoxic metabolites (Yu and Powles, 2014) or aphids, for which the resistance to the insecticide spirotetramat has recently been ascribed to UGTs (Pan et al., 2020). In the model nematode *Caenorhabditis elegans*, a resistance-related UGT (ugt22) has been reported which is responsible for a higher tolerance toward ABZ (Fontaine and Choe, 2018; Stasiuk et al., 2019). In *H. contortus*, several studies have already confirmed the potential of UGTs in the resistance mechanisms; higher formation of glycosides (Vokral et al., 2013; Stuchlikova et al., 2018), and higher expression of several UGTs in adults of resistant isolates (Matouskova et al., 2018).

**FIGURE 1:**
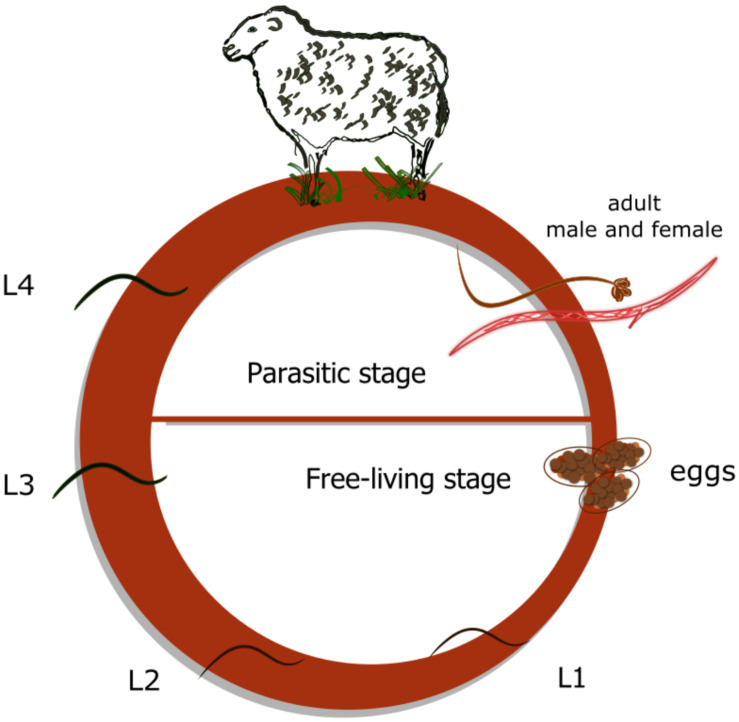
*Haemonchus contortus* -life cycle. Three non-parasitic (free-living) stages: eggs, first stage larvae (L1) and second stage larvae (L2); free-living infective third stage larvae (L3); parasitic (blood sucking) fourth stage larvae (L4); and dimorphic adults.

We have previously characterized the UGT family in *H. contortus*, including its nomenclature (approved by the UGT nomenclature committee), phylogeny, and expression in adults – the parasitic life stage (Matouskova et al., 2018). However, nothing is known about the metabolism of anthelmintic drug ABZ or expression of UGTs in the juvenile stages – the free living stages. Unlike many other parasites, the life cycle of *H. contortus* is quite simple and does not involve any intermediate host (Figure 1). Under optimal conditions, first-stage larvae (L1s) hatch from eggs within 24–72 h and continue molting through second-stage larvae (L2s) into the infective third-stage larvae (L3s). L3s are protected by two layers of cuticle and actively climb up blades of grass waiting to be eaten by a ruminant. Subsequently, sudden and substantial changes in the environment (heat shock and increased carbon dioxide levels) that occur with ingestion trigger the exsheathment of L3s (Bekelaar et al., 2018), which then mature through the fourth-larval stage (L4s) into dimorphic adults. Both L4s and adults reside in the abomasum, where they feed on blood, thus placing a severe burden on the host (Harder, 2016).

Since the development from egg to L3s occurs within feces on the pasture, these stages can get in contact with many xenobiotics, including anthelmintics, and their metabolites. The treated animal excretes ABZ and ABZSO is substantial amounts, especially within the first 24 h (Prchal et al., 2016). However, the drug is quite persistent and stays on the pasture for a long time (Porto et al., 2020). If the treated animal is left freely on pasture, the juvenile stages can easily get into contact with the drug, which may contribute to resistance development. We, therefore, decided to study the expression of UGTs and the metabolism of ABZ and ABZ-sulfoxide (ABZSO; the primary active ABZ metabolite) in the eggs, L1s and L3s larvae of *H. contortus*. We have also compared the formation of ABZ metabolites and selected UGT transcripts between a sensitive isolate (ISE) and resistant isolates (IRE and multi-resistant WR). Furthermore, we explored the inducibility of UGTs by ABZ and ABZSO in *H. contortus* juvenile stages.

## Materials and Methods

### *H. contortus* Strains and Culturing

In this study, juveniles (non-parasitic stages) of three isolates of *H. contortus* were used: (i) ISE: the inbred susceptible-Edinburgh strain (MHco3; Roos et al., 2004), (ii) IRE: the inbred resistant-Edinburgh strain (MHco5), and (iii) WR: the White River multidrug resistant strain (MHco4). The juveniles were obtained from previously infected lambs with 6,000 L3s of the *H. contortus* strains separately. The nematode eggs were collected by differential sieving through three stacked sieves as described by Varady et al. (2007) and recovered with a sucrose flotation technique followed by washing in tap water. The L1s were produced from isolated eggs cultivated in tap water at 27°C for 48 h, while the L3s were cultivated from eggs in humidified feces from infected sheep at 27°C for 1 week (Nguyen et al., 2019).

### Experimental Design: Exposure of the Nematodes to Anthelmintics

For the transcriptional analysis, 100 thousand freshly isolated eggs, 100 thousand freshly cultivated L1s, and 30 thousand L3s of *H. contortus* ISE, IRE, and WR strain per sample were used. Four biological replicates of each stage and strain were placed directly into TriReagent^®^ (Molecular Research Centre, OH, United States) for immediate RNA isolation or stored in −80°C for later use. Four more biological replicates of all stages from the ISE and IRE strain were stimulated without (0.1% DMSO control) or with 0.5 μM ABZ (Sigma-Aldrich, Prague, Czechia) and 0.5 μM ABZSO (Sigma-Aldrich, Prague, Czechia) for 4 h, both drugs were prepared as 0.5 mM stock solutions in DMSO (Sigma-Aldrich, Prague, Czechia) prior to each experiment. Furthermore, the L3s were also exposed to two higher drug concentrations: 1 μM and 10 μM ABZ and/or ABZSO for 4 h. After incubations, all samples were placed into 1 mL of TriReagent^®^ for immediate RNA isolation or stored in −80°C for later use.

For the analysis of ABZ metabolism, 100 thousand freshly isolated eggs, 60 thousand freshly cultivated L1s, and 30 thousand L3s of *H. contortus* ISE or IRE strain per sample were used. All the juveniles were incubated in 4 mL of medium (sterile tap water) with 0.5 μM ABZ or 0.5 μM ABZSO for 24 h in three biological replicates. The concentrations were chosen based on the egg hatch test (Supplementary Material) and on previous IC_50_ estimation by larval development assay (Prchal et al., 2016). The ISE and IRE L3s were also incubated with 1 μM and 10 μM ABZ or ABZSO, as they are protected by three layers of cuticle and can tolerate higher concentrations. The final concentration of DMSO in water was 0.1% (v/v). After a 24 h incubation, the medium from the juveniles was placed into plastic tubes. The juveniles were washed three times with sterile tap water and were also transferred into the plastic tubes. The samples were frozen and stored at −20°C for later use. Chemical blanks (tap water with anthelmintics, without juveniles) and biological blank samples (medium with juveniles and 0.1% DMSO, without anthelmintics) were prepared in the same way.

### Transcriptional Analysis

Total RNA from all samples was extracted using TriReagent^®^ according to the manufacturer’s protocol, following previous homogenization of the samples in the FastPrep-24 5G Homogenizer (MP Biomedicals, France) using 0.2 mm ceramic beads with an additional one 0.5 mm metal bead for four subsequent 30 s steps (6 m.s^–1^) to maximize the cell breakage of eggs and larvae. The integrity and concentrations of RNA was analyzed using the Agilent 2100 Bioanalyzer on RNA Nano chips (Agilent Technologies, CA, United States) and measured spectrophotometrically with the NanoDrop ND-1000 UV – Vis Spectrophotometer (Thermo Fisher Scientific, MA, United States) at a wavelength of 260 and 280 nm, respectively. 4 μg of extracted RNA were treated with DNase I (NEB, United Kingdom) followed by reverse transcription using 0.5 μg RNA, random hexamers and Protoscript^®^ II Reverse Transcriptase (NEB, United Kingdom; in 20 μL reaction mixture) according to the manufacturer’s protocol.

The synthetized cDNA was diluted to a final concentration of 12.5 ng/μL DNA and stored at −20°C or used for qPCR analyses performed in the QuantStudioTM 6 Flex Real-Time PCR System (Applied Biosystems, CA, United States) with SYBR Green I detection as described previously (Kellerova et al., 2019) with the difference in the final volume 8 μL per well on 384-well Plates. A combination of two reference genes out of the three genes tested, RNA polymerase II (large subunit gene; *ama*), glyceraldehyde-3P-dehydrogenase (*gpd*) and/or the nuclear-cap binding protein subunit 2-like (*ncbp*), were used for normalization of the qPCR assay (Lecova et al., 2015). The 31 UGT primer sets were adopted from our previous studies (Lecova et al., 2015; Matouskova et al., 2018; UGT366B1 and B2 were tested by common primer set, since due to very similar sequences it is impossible to design well functional separate primer sets). The primer sequences, amplicon sizes, and efficiencies are listed in Supplementary Table 1.

### Metabolism Analysis

The nematodes were repeatedly homogenized six-times for 30 s in cooled 0.1 M phosphate buffer (pH 7.4) using homogenizer the Fastprep-24 5G, then centrifuged for 5 min at 3,000 × *g*. The protein concentration in the nematode homogenates was measured using a bicinchoninic acid method (Sigma-Aldrich, Prague, Czechia) according to the manufacturer’s protocol. Supernatants of the homogenates as well as medium samples were extracted using solid-phase extraction (SPE) columns as described previously (Stuchlikova et al., 2013). The dry samples were quantitatively reconstituted in the acetonitrile/water mixture (30:70, v/v) using sonication and a vortex for 5 min. One microliter of the reconstituted samples was injected into the UHPLC-MS system. UHPLC (Nexera; Shimadzu, Kyoto, Japan) was optimized using the Zorbax RRHD Eclipse Plus C18 column 150 × 2.1 mm, 1.8 μm (Agilent Technologies, Waldbronn, Germany) at a temperature of 40°C, a flow rate 0.4 mL/min and injection volume of 1 μL. The mobile phase consisted of water (A) and acetonitrile (B), both with the addition of 0.1% formic acid (MS grade). The linear gradient was as follows: 0 min – 15% B, 8 min – 40% B, and 10 min – 95% B followed by 1 min of isocratic elution. The QqQ mass spectrometer (LC-MS-8030 triple quadrupole mass analyzer; Shimadzu, Kyoto, Japan) was used with the following tuning parameters settings: capillary voltage 4.5 kV, heat block temperature 400°C, DL line temperature 250°C; drying gas flow rate 12 L/min and 2.5 L/min of nebulizing gas, respectively. The mass spectrometer was operated in positive ion MRM (multiple reaction monitoring) mode. Argon was used as collision gas for the MS/MS experiments.

The optimization of mass spectrometer parameters and MRM transitions of ABZ, ABZ-SO, ABZ-SO2, and mebendazole (MEB) was accomplished using analytical standards dissolved in acetonitrile/water (30/70, v/v). MRM transitions of the metabolites were used according to the previous publication by Stuchlíková et al. (2018).

The standards of the potential metabolites were generally not commercially available, and they were not prepared due to the difficulties associated with their synthesis. For this reason, the amounts of metabolites were semi-quantified using a ratio of peak areas of the metabolites with the area of the internal standard (IS) peak (MEB). In the nematode homogenates, these ratios were normalized per milligram of total protein.

### Data Analysis

The data for statistical analysis were expressed as the mean ± S.E.M. (3–4 biological replicates of each sample). The normality distribution was confirmed using Shapiro–Wilk normality test (alpha = 0.05). A statistical significance of gene expression differences between sensitive strain and resistance strains was evaluated on normalized data (the ISE set to 1) using Multiple *t*-tests with Holm-Sidak comparison correction. The inducibility results were analyzed likewise, where the controls were set to 1. In the metabolism analysis the statistical differences among the stages was for each metabolite analyzed using one-way ANOVA followed by Tukey’s multiple comparison test. The statistical differences in metabolism between strains were evaluated using Multiple *t*-tests with Holm-Sidak comparison correction. To enable statistical analysis of the metabolites that were not detected (n.d.), the cut off limit for the area under the peak was used instead. All statistical tests were performed using GraphPad Prism software 8.4.3 (GraphPad Prism^®^, United States), with differences considered significant at *P* < 0.05.

## Results

### Comparison of the Constitutive Expression of UGT in Juvenile Stages of ISE *H. contortus* (Eggs, L1s, L3s)

**FIGURE 2:**
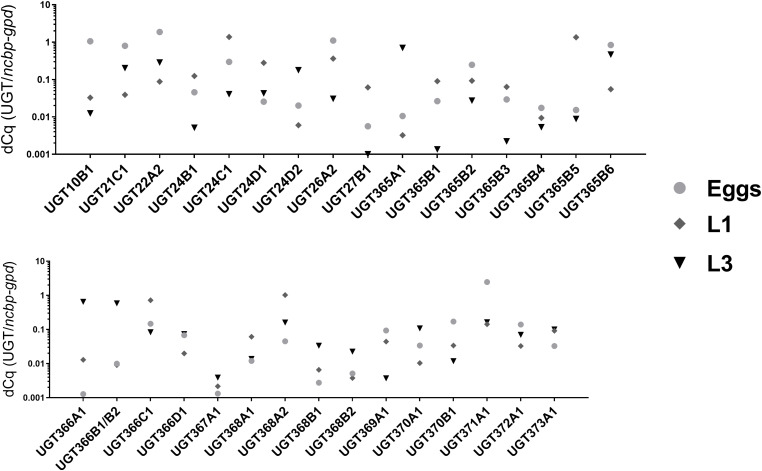
Relative abundancies of UGT genes from three juvenile (non-parasitic) stages: eggs, first stage larvae (L1s) and the third stage larvae (L3s). For each UGT the mean of relative expression is displayed as ΔCq, normalized to geometric mean of *ncbp* and *gpd*hebib.

The transcript level of 32 UGT genes were tested using qPCR, with the relative quantity (ΔCq, normalized to the geometric mean of the two reference genes *ncbp* and *gpd*) of each UGTs were compared among three juvenile (free-living) stages: eggs, L1s, and L3s (Figure 2). The comparison among the eggs and larval stages showed differences for all transcripts, suggesting UGTs’ individual roles in each life stage. Nine UGTs were predominantly expressed in the eggs; ten and six UGTs showed elevated transcription levels in the L1s and L3s, respectively. Supplementary Figure 1 displays as well the expression in females and males to draw the comparison with adult stages measured previously (Matouskova et al., 2018).

### Constitutive UGT Expression in Juvenile Stages of Different *H. contortus* Strains

**FIGURE 3:**
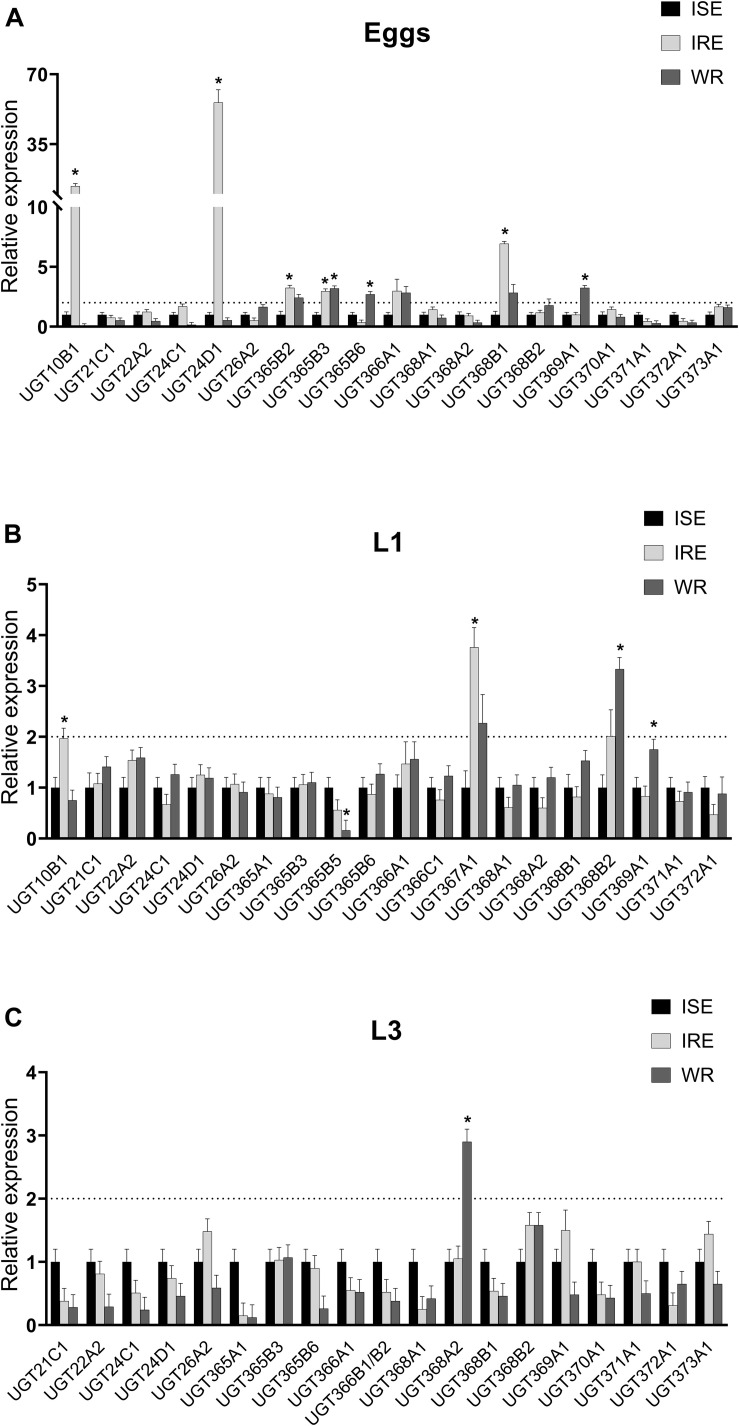
Comparison of constitutive UGT mRNAs expression in three juvenile (non-parasitic) stages: eggs **(A)**, first stage larvae (L1s; **B**) and the third stage larvae (L3s; **C**) of three different strains drug-susceptible ISE and two drug-resistant IRE and WR. The UGT mRNA expression of resistant strains is relative to the levels of ISE strain, which was normalized to 1. The mRNA fold changes were calculated using geometric mean of two reference genes (*gpd*, *ama*). Data represent the mean ± S.E.M. (*N* = 4), *indicates significant difference between ISE and IRE or WR, *P* < 0.05.

Constitutive expressions of selected UGTs (in eggs, L1s, and L3s) in three *H. contortus* strains were tested (Figure 3). Each tested UGT in two drug-resistant strains IRE and WR was relatively compared to the drug-susceptible ISE strain. In the eggs (Figure 3A), 7 UGT genes out of 19 tested, selected based on previous report (Matouskova et al., 2018) and predominant expression in respective stage, showed significant overexpression at least in one resistant strain in comparison to the susceptible one. The highest difference in transcription level was observed for UGT10B1 (13-fold), UGT24D1 (55-fold), and UGT368B1 (6-fold) in the IRE strain. Only one transcript (UGT365B3) showed a higher level in the eggs of both resistant strains IRE and WR. In the L1s, 4 out of the 20 tested UGTs were overexpressed in one of the resistant strains (Figure 3B). Only UGT368A2 showed significantly higher transcription level in the L3s of the resistant strain WR.

### Transcription Response of UGTs to 4-h Exposure to ABZ and ABZSO in Juvenile Stages of *H. contortus* Sensitive Strain

**FIGURE 4:**
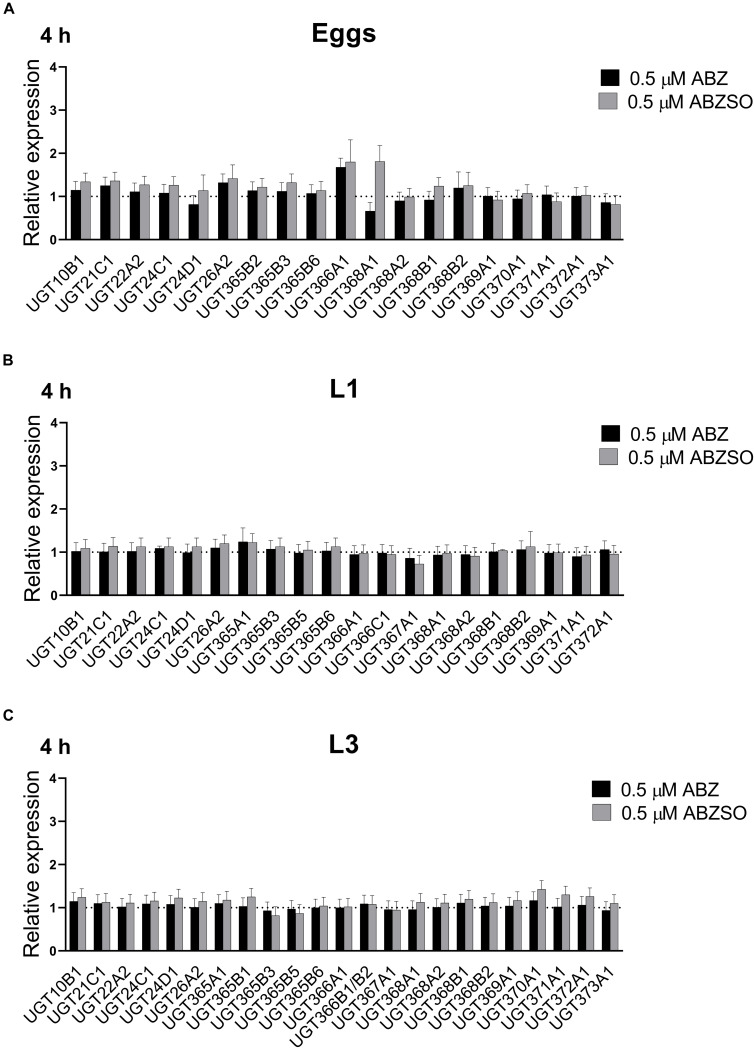
Expression of UGT mRNAs in eggs **(A)**, L1s **(B)**, and L3s **(C)** juvenile stages of *H. contortus* susceptible strain ISE after stimulation with 0.5 μM ABZ and ABZSO concentration. The mRNA expression levels were normalized to the levels of controls (non-stimulated nematodes) displayed as dotted line (=1). The mRNA fold changes were calculated using geometric mean of two reference genes (*gpd*, *ama*). Data represent the mean ± S.E.M. (*N* = 4).

The juvenile stages (non-parasitic) stages of ISE *H. contortus* were exposed to ABZ and ABZSO. 4-h exposure of 0.5 μM ABZ and 0.5 μM ABZSO did not cause any significant changes in any of the stages tested (Figure 4). Additionally, the L3s were exposed to two higher concentrations of ABZ and ABZSO (1 μM and 10 μM) for 4 h. Interestingly, both drugs only at 1 μM concentration greatly enhanced the expression of UGT368A2 (7- and 3-fold induction) and UGT371A1 (9- and 7-fold induction by ABZ and ABZSO, respectively). None of these UGT changes were observed with the higher concentration (10 μM; Supplementary Figure 2).

### The Comparative Analysis of ABZ and ABZSO Metabolism in Juvenile Stages of *H. contortus* ISE and IRE Strain

**FIGURE 5:**
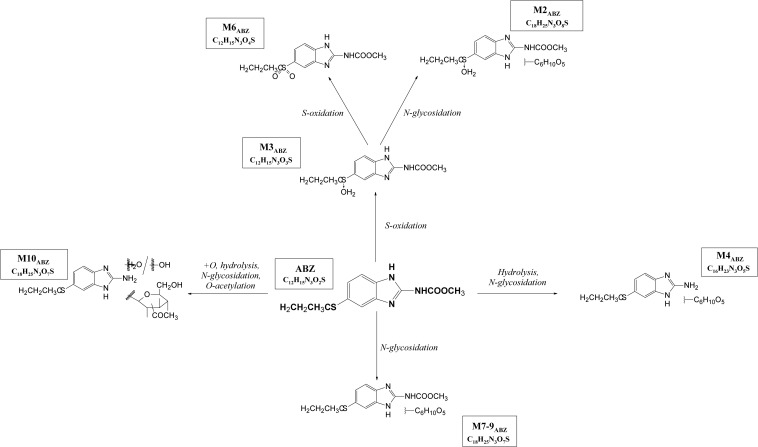
The proposed metabolic pathway of albendazole (ABZ) in *H. contortus* juvenile stages.

With the aim of determining whether the juvenile stages of *H. contortus* are capable of anthelmintics biotransformation, the metabolism of ABZ and ABZSO were studied in the eggs, L1s and L3s of *H. contortus* susceptible strain ISE. Furthermore, the level of metabolites formation was compared in *H. contortus* benzimidazole resistant strain IRE. The eggs and larvae were incubated in medium without any drug (0,1% DMSO controls) and with ABZ or ABZSO at the concentration 0.5 μM for 24 h. In addition, the L3s were incubated with two higher ABZ and ABZSO concentrations; 1 μM and 10 μM (Supplementary Material). Qualitative and semi-quantitative analyses of the ABZ metabolites in homogenate and in medium from the eggs and larvae of both strains were performed using UHPLC/MS. The metabolites were identified and named based on their retention times and fragmentation ions as described previously in adults (Stuchlikova et al., 2018; Figure 5, Table 1). The detected ABZ metabolites were mostly formed via S-oxidation resulting in ABZSO (M3_ABZ_) and 2^∗^S-oxidation forming ABZSO2 (M6_ABZ_) in the juvenile stages, as in the adults (Stuchlíková et al., 2018; Kellerová et al., 2020).

**TABLE 1:** Biotransformation of ABZ in *H. contortus* juvenile stages – the metabolites detected by UHPLC-MS/MS.

**Metabolite designation**	**t_*R*_ [min]**	**Theoretical *m/z* values of [M + H]^+^ ions**	**Elemental composition**	**Description of metabolite formation**		**Product ions of [M + H] +, *m/z***	**Metabolite**
				**Phase I**	**Phase II**		
M2_*ABZ*_	2.5	444.14	C_18_H_25_N_3_O_8_S	S-oxidation	*N*-glycosidation	282, 240, 208	*N*-glycoside of ABZSO
M3_*ABZ*_	3.4	282.09	C_12_H_15_N_3_O_3_S	S-oxidation	–	240, 208, 191,159	ABZSO
M6_*ABZ*_	5.2	298.09	C_12_H_15_N_3_O_4_S	2*S-oxidation	–	266, 224, 159	ABZSO2
M7_*ABZ*_	5.6	428.15	C_18_H_25_N_3_O_7_S	–	*N*-glycosidation	266, 234	*N*-glycoside of ABZ
M8_*ABZ*_	6.1	428.15	C_18_H_25_N_3_O_7_S	–	*N*-glycosidation	266, 234	*N*-glycoside of ABZ
M9_*ABZ*_	6.6	428.15	C_18_H_25_N_3_O_7_S	–	*N*-glycosidation	266, 234	*N*-glycoside of ABZ
M10_*ABZ*_	6.0	428.15	C_18_H_25_N_3_O_7_S	+O, hydrolysis	Glycosidation, O-acetylation	208	Acetylglucoside of ABZ
ABZ (parent drug)	7.4	266.10	C_12_H_15_N_3_O_2_S	–	–	234	

**FIGURE 6:**
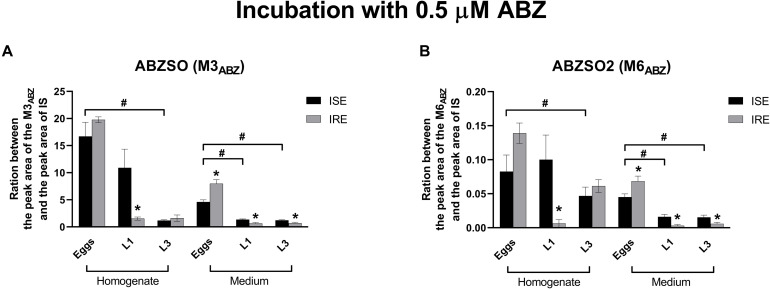
Changes in the relative amount of the two ABZ metabolites **(A)** M3_ABZ_ (ABZSO) and **(B)** M6_ABZ_ (ABZSO2) in juvenile’s homogenates of the susceptible ISE strain and resistant IRE strain after 0.5 μM ABZ incubation. The data represent the mean ± S.E.M. (*N* = 3). IS = internal standard. *indicates significant difference between ISE and IRE; and ^#^indicates the significant differences between stages in ISE strain, *P* < 0.05.

**FIGURE 7:**
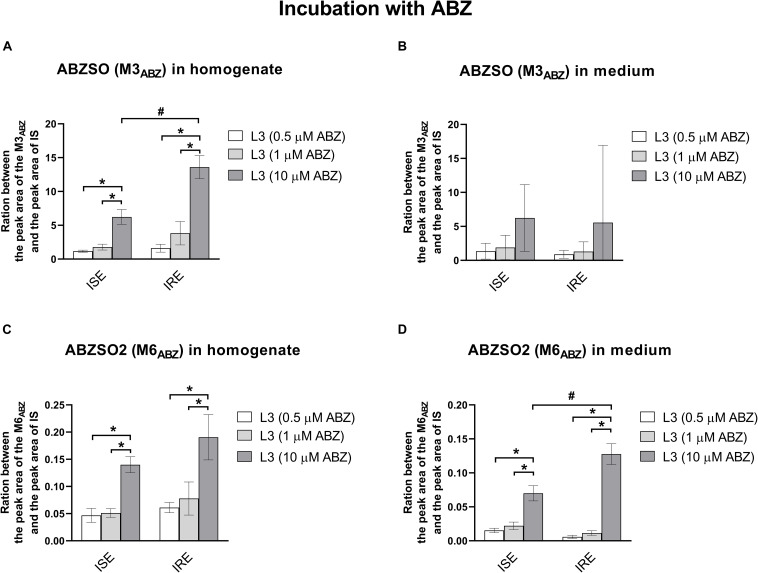
The comparison of the relative amount of the two ABZ metabolites **(A,B)** M3_ABZ_ (ABZSO) and **(C,D)** M6_ABZ_ (ABZSO2) in homogenates and medium (respectively) of the L3s susceptible ISE strain and resistant IRE strain incubated in three different concentration of ABZ (0.5 μM, 1 μM, and 10 μM). The data represent the mean ± S.E.M. (*N* = 3). IS = internal standard, ^#^indicates significant differences between ISE and IRE for respective concentration; and *indicates significant differences between different concentrations, *P* < 0.05.

Albendazole-sulfoxide (M3_ABZ_) and ABZSO2 (M6_ABZ_) formation is higher in the eggs and L1s (ISE strain only) than in the L3s (as detected in homogenates); significantly lower amounts of both metabolites were detected in the medium surrounding the L1s and L3s (Figure 6). Significant differences in the metabolism between the ISE and the IRE strain, were found. Although both eggs transformed ABZ to M3_*ABZ*_ and M6_*ABZ*_ to a similar extent, the IRE eggs were able to eliminate both oxidized metabolites into the medium much more effectively. On the contrary, significantly fewer metabolites were found in the medium surrounding the IRE L1s and L3s. Because the infectious *H. contortus* stage L3s is able to tolerate higher ABZ concentration, the L3s were exposed to two higher ABZ concentrations, as shown in Figure 7, causing a greater formation of both ABZ metabolites (M3 and M6). Significantly more M3_ABZ_ was detected in the homogenate of the IRE L3s, but not in the medium. On the other hand, M6_ABZ_ was found at comparable amounts in both homogenates, although the IRE L3s were able to eliminate this metabolite into the medium much more efficiently.

**FIGURE 8:**
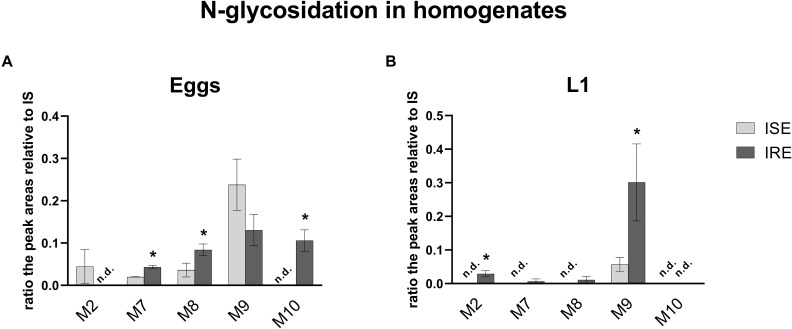
in the comparison of the relative amount of the five N-glycosides of ABZ in **(A)** eggs and **(B)** L1 homogenates of the susceptible ISE strain and resistant IRE strain. The data represent the mean ± S.E.M. (*N* = 3). IS = internal standard, n.d. = not detected. *indicates significant difference between ISE and IRE, *P* < 0.05.

Due to the Phase II biotransformation, four glycosidated metabolites (three N-glycosides of ABZ; M7_ABZ_, M8_ABZ_, M9_ABZ_, and one N-glycoside of ABZSO; M2_ABZ_) were formed in the ISE eggs homogenates. In IRE eggs, two of the ABZ N-glycosides (M7_ABZ_ and M8_ABZ_) were significantly higher and acetylglucoside of ABZ (M10_ABZ_) was detected, instead of M2_ABZ_ (Figure 8). In both ISE strain larval stages, only one additional *N*-glycosidated metabolite (M9_ABZ_) was detected besides M3_ABZ_ and M6_ABZ_ (Table 2). On the other hand, the IRE L1s were able to transform ABZ into three additional N-glycosides (M2_ABZ_, M7_ABZ_, and M8_ABZ_), suggesting a higher UGT enzyme activity in the IRE strain than in the ISE.

**TABLE 2:** Presence (+) or absence (−) of ABZ metabolites in homogenates and medium of *H. contortus* juvenile stages from ISE and IRE strains.

**Metabolite designation**	**Homogenate**										**Medium**									
	**ISE**					**IRE**					**ISE**					IRE				
	**Eggs (0.5 μM)**	**L1 (0.5 μM)**	**L3 (0.5 μM)**	**L3 (1 μM)**	**L3 (10 μM)**	**Eggs (0.5 μM)**	**L1 (0.5 μM)**	**L3 (0.5 μM)**	**L3 (1 μM)**	**L3 (10 μM)**	**Eggs (0.5 μM)**	**L1 (0.5 μM)**	**L3 (0.5 μM)**	**L3 (1 μM)**	**L3 (10 μM)**	**Eggs (0.5 μM)**	**L1 (0.5 μM)**	**L3 (0.5 μM)**	**L3 (1 μM)**	**L3 (10 μM)**
M2_*ABZ*_	+	–	–	–	–	–	+	–	–	–	–	–	–	–	–	–	+	–	–	–
M3_*ABZ*_	+	+	+	+	+	+	+	+	+	+	+	+	+	+	+	+	+	+	+	+
M6_*ABZ*_	+	+	+	+	+	+	+	+	+	+	+	+	+	+	+	+	+	+	+	+
M7_*ABZ*_	+	–	–	–	–	+	+	–	–	–	–	+	–	–	–	–	+	–	–	–
M8_*ABZ*_	+	–	–	–	–	+	+	–	–	–	+	–	–	–	–	–	+	–	–	–
M9_*ABZ*_	+	+	–	–	+	+	+	–	–	–	–	–	–	–	–	–	+	–	–	–
M10_*ABZ*_	–	–	–	–	–	+	–	–	–	–	–	–	–	–	–	–	–	–	–	–
ABZ	+	+	+	+	+	+	+	+	+	+	+	+	+	+	+	+	+	+	+	+

Only two metabolites of ABZSO (or ricobendazole), an anthelmintically active metabolite of ABZ, were formed; M6_*ABZ*_ (ABZSO2) via S-oxidation and ABZ via the reduction of sulfoxide (-O; Supplementary Tables 2, 3) in both studied strains. After incubation with ABZSO, the eggs effectively eliminated the ABZSO2 (M6_ABZ_) metabolite into the media, since no ABZSO2 was detected in the homogenate. In the larval stages, ABZSO2 was detected in both the media and homogenates (Supplementary Figure 3). Only the IRE L3s formed significantly more M6_ABZ_ than ISE L3s in the homogenates, whereas the IRE L1s transported less M6_ABZ_ than the ISE L1s in the medium. Similarly, as in ABZ incubation, the higher ABZSO concentration caused a greater amount of formed M6_ABZ_, both in the homogenates and media (Supplementary Figure 4).

## Discussion

Generally, glycosidation is a strategy for minimizing the accumulation of potentially toxic chemicals in cellular membranes by facilitating their excretion. Therefore, UGTs provide a protective interface between the organism and an environment rich with harmful xenobiotics and pathogens along with other unfavorable conditions (Hu et al., 2019). Free-living stages of *H. contortus* may be in contact with various xenobiotics including the anthelmintics excreted from treated animals (Prchal et al., 2016). After deworming pharmacotherapy, animals are usually left free to roam in pastures, which leads to an increased contamination of the environment with the drugs and their metabolites. In this way, the juvenile stages of helminths in such pastures are permanently exposed to a sublethal concentration of the drug, which might promote drug-resistance development upon reinfection. We hypothesize that UGTs in the juvenile stages of *H. contortus* may well represent not only an important defense system against harmful anthelmintics, but also the means of helping to develop drug-resistance.

In previous studies, we measured the expression levels of all identified UGTs in the *H. contortus* genome (Laing et al., 2013; Matouskova et al., 2018). The levels of most of the UGTs during development well correlate when compared to the transcriptomic data (TPMs – transcript per million) from Laing et al. (2013; Supplementary Figure 5). Overall variation in the transcriptional pattern among life stages is presumed, as different UGTs can have various roles in a particular life stage. Surprisingly, nine of the UGT transcripts are expressed in highest amounts in eggs than other life stages. Since eggs do not take up any external supplement, transcription activity mainly focuses on development (Laing et al., 2013; Schwarz et al., 2013). Interestingly, three of the genes highly expressed in eggs (UGT10B1, 22A2, and 26A2) belong to a group that have single-membered homologues in *C. elegans*, suggesting an “old” role for these enzymes (Matouskova et al., 2018). Ten UGTs are expressed mostly in the L1s that may correspond to active feeding (Laing et al., 2013), three of which belong to UGT24 family and three to UGT365B family, which is the most expanded family in *H. contortus*. On the other hand, only five genes show the highest expression in L3s; specifically, UGT365A1 has a much higher expression (more than a 100-fold difference) compared to all other life stages. Curiously, when compared to expression levels in adults, which was measured in our previous study (Matouskova et al., 2018; Supplementary Figure 1), most of the UGTs showed a higher expression in juvenile stages, with only UGT365B4, UGT368B1, and UGT373A1 having a highest expression in adult males, and UGT366D1 and UGT369A1 in adult females (the stages living within the host and feeding on blood).

To reveal resistance-related changes in UGTs expression, we compared the levels of selected UGTs in a drug-sensitive strain ISE and drug-resistant strains IRE and WR (The resistance of the IRE strain was confirmed by egg hatch test as shown in Supplementary Figure 6). The results showed some interesting variations. In the eggs and L1s, different genes were upregulated in the resistant strains than were identified previously in the adults, e.g., UGT10B1 and UGT24D1 in the IRE eggs, and UGT367A1 in both L1s. Only UGT368B2, which showed the highest difference between the sensitive strain and both resistant strains in the adults, also showed a higher expression in both resistant strains in the L1s and L3s, a finding which warrants further investigations into the function of this UGT (Matouskova et al., 2018).

The UGTs inducibility by sublethal dose of ABZ or ABZSO was not proved in free-living stages of *H. contortus.* Only, 1 μM ABZ (and ABZSO) significantly upregulated two UGTs in the L3s, while 10 μM drug concentrations had no effect on UGTs expression, possibly due to toxicity. These discrepancies clearly show the importance of testing different concentrations, not only the very high concentration typically used for metabolism studies. In *C. elegans*, 4-h ABZ exposure (1.13 mM) increased the expression of eight UGTs (Stasiuk et al., 2019). When Stasiuk et al. (2019) tested ABZ-mediated induction of UGTs in *H. contortus* adults, no transcriptional response was detected. However, this may be due to the very high ABZ concentration used (Stasiuk et al., 2019). On the other hand, adults exposed to very low sublethal concentrations showed an increase in the transcription of several UGTs, including UGT368B2 (Kellerová et al., 2020).

Because glucose conjugation (hexose) represents an important biotransformation of anthelmintics in *H. contortus* adults (Vokral et al., 2013; Stuchlíková et al., 2018; Stasiuk et al., 2019), we were interested in the biotransformation pathway of anthelmintics in the juvenile stages of *H. contortus*. The biotransformation study of ABZ and ABZSO showed that the most dominant process in the juvenile stages was S-oxidation, with ABZ metabolized at a high rate into M3_ABZ_ (ABZSO) and further to M6_ABZ_ (ABZSO2).

Concerning glycosidation, several ABZ-glycosides and ABZSO-glycosides were formed in a reasonable amount in the *H. contortus* juvenile stages, which proved UGTs participation in anthelmintics detoxification in eggs and larvae similarly as in adults (Stuchlíková et al., 2018). In the resistant L1s, three of the glycosidated metabolites were formed in higher amount than in sensitive one. If we compare the number of metabolites detected in larval stages among the strains, in the ISE there were only three glycosidated metabolites detected, while in the IRE three more glycosidated metabolites were found. This corresponds with the biotransformation of ABZ among adult worms of drug-susceptible and drug-resistant *H. contortus* strains, previously reported by Stuchlíková et al. (2018). In this study, the adults of the resistant IRE strain showed a greater ability to deactivate anthelmintics via metabolism than did the adults of the susceptible ISE strain by forming more metabolites of both biotransformation phases, e.g., oxidation, reduction, hydrolysis and glycosidation or acetylation (Stuchlíková et al., 2018). Interestingly, only IRE eggs were able to form M10_ABZ_ (acetylglucoside of ABZ), which was detected in ISE adults in a previous study (Kellerová et al., 2020). On the other hand, the metabolite M2_ABZ_ (*N*-glycoside of ABZSO) was found only in the ISE eggs homogenate and IRE L1 homogenate and in the medium. Seven metabolites of ABZSO (ricobendazole, the main anthelmintically active albendazole metabolite), were reported in adults (Stuchlíková et al., 2018), however, in this study only two metabolites were formed in the juvenile stages; M6_*ABZ*_ (albendazole sulfon) via *S*-oxidation and ABZ via the reduction of ABZSO.

Altogether, our results showed UGTs constitutive expression, possible resistance-related changes and the participation in ABZ detoxification in the individual juvenile stages of *H. contortus*. However, it is difficult to estimate which UGT might be important for xenobiotic metabolism only from transcription studies. Further, since functional testing and/or gene silencing remains difficult in *H. contortus* (Britton et al., 2012), some speculation based on our results may be useful, especially in connection to the potential players in resistance. The UGT371A1 was upregulated by 1 μM ABZ and ABZSO in L3s, as well as in susceptible strain ISE exposed to sub-lethal doses of ABZ and ABZSO (Kellerová et al., 2020). It also showed a higher constitutive expression in resistant males (Matouskova et al., 2018). The UGT368B2 is overexpressed in resistant strains in both adults (Matouskova et al., 2018) and in larvae stages. Furthermore, it is inducible by sublethal concentrations in adults (Kellerová et al., 2020).

In conclusion the biotransformation in juvenile stages of *H. contortus* is similar to well described metabolism in adults. UGTs proved to be important enzymes for detoxification of albendazole and furthermore, the observed differences in resistant strain warrant further investigation into the function of these enzymes.

## Data Availability Statement

The raw data supporting the conclusions of this article will be made available on request from the corresponding author.

## Ethics Statement

The animal study was reviewed and approved by Ethics Committee of the Czech Ministry of Education, Youth and Sports (Protocol MSMT-25908/2014-9).

## Author Contributions

PK, PM, and LS contributed to study design. DD and LN prepared the juvenile stages. PK, DD, LN, KS, and MN carried out the analysis. PK, MN, LR, and PM contributed to data analysis. PK drafted the manuscript. PM, LN, and LS edited the final version. All authors approved the final version of the manuscript.

## Conflict of Interest

The authors declare that the research was conducted in the absence of any commercial or financial relationships that could be construed as a potential conflict of interest.
