# Supplementary material for: UDP-Glycosyltransferases and Albendazole Metabolism in the Juvenile Stages of Haemonchus contortus
Source: Front Physiol. 2020 Nov 26;11:594116. doi: 10.3389/fphys.2020.594116 (PMC7726322; doi:10.3389/fphys.2020.594116)
Supplement: Supplementary file 1 [file Table_1.docx]

Supplementary Material

**Supplementary Figure 1**: Relative abundances of UGT genes compared with adults. Three juvenile (non-parasitic) stages: eggs, first stage larvae (L1s), and the third stage larvae (L3s) female and male (red and blue lines)^≠^; of susceptible ISE strain. For each UGT the mean of relative expression is displayed as ΔCq, normalized to the geometric mean of *ncbp* and *gpd*, N ≥ 3.

**
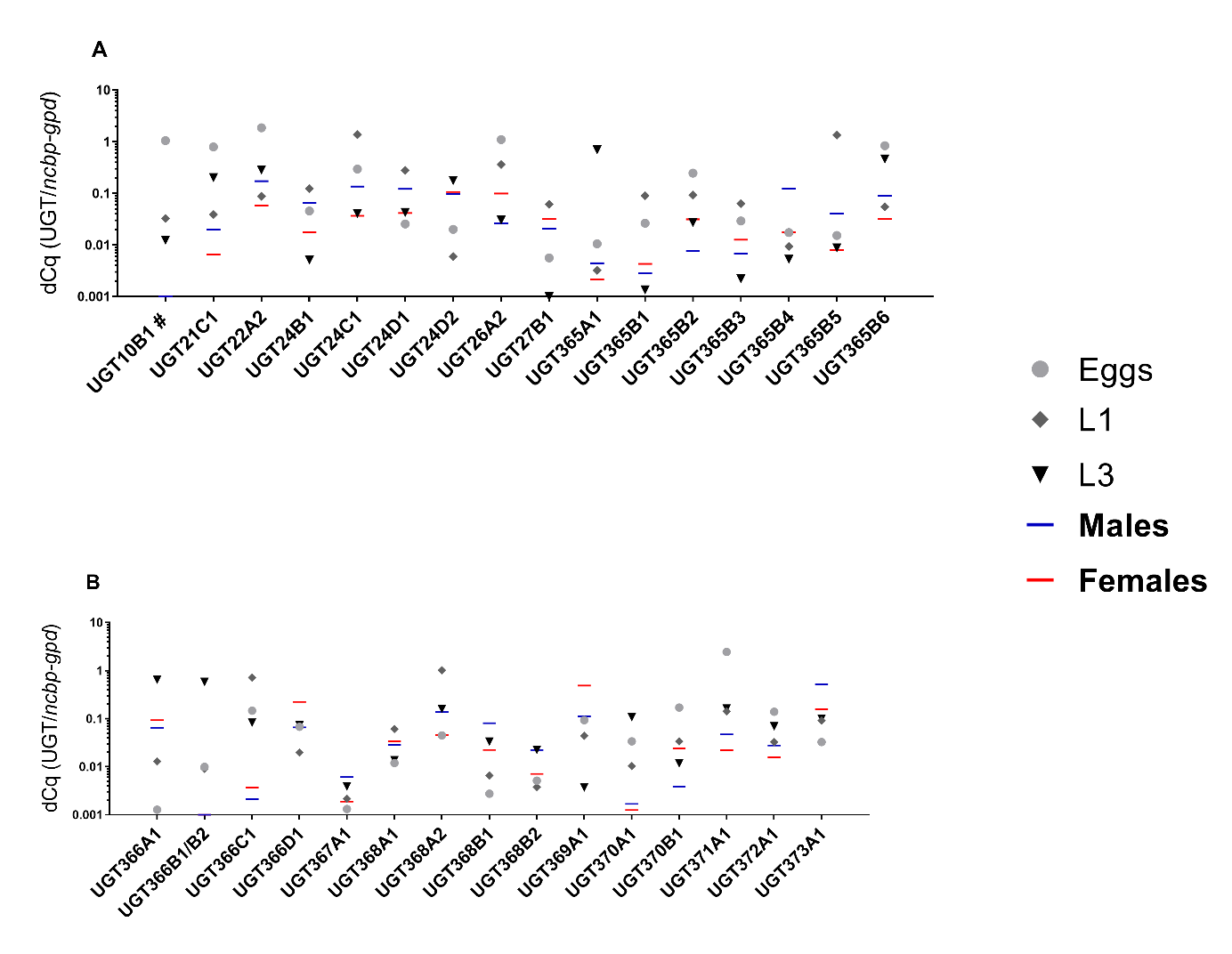
**

^#^ UGT10B1 not detected in adult males and only traces in adult females

≠ The data corresponding to femal and male adults was adopted from. Matouskova, P., et al 2018; doi: 10.1016/j.ijpddr.2018.09.005

**Supplementary Figure 2**. Expression of UGT mRNAs in L3 of *H. contortus* susceptible strain ISE after stimulation with 1 µM and 10 µM ABZ and ABZSO concentration (a and b, respectively). The mRNA expression levels were normalized to the levels of controls (non-stimulated nematodes) displayed as a dotted line (= 1). The mRNA fold changes were calculated using geometric mean of two reference genes (*gpd*, *ama*). Data represent the mean ± S.E.M. (N=4). * indicates a significant difference between non-stimulated and ABZ- or ABZSO-stimulated stages, P < 0.05.


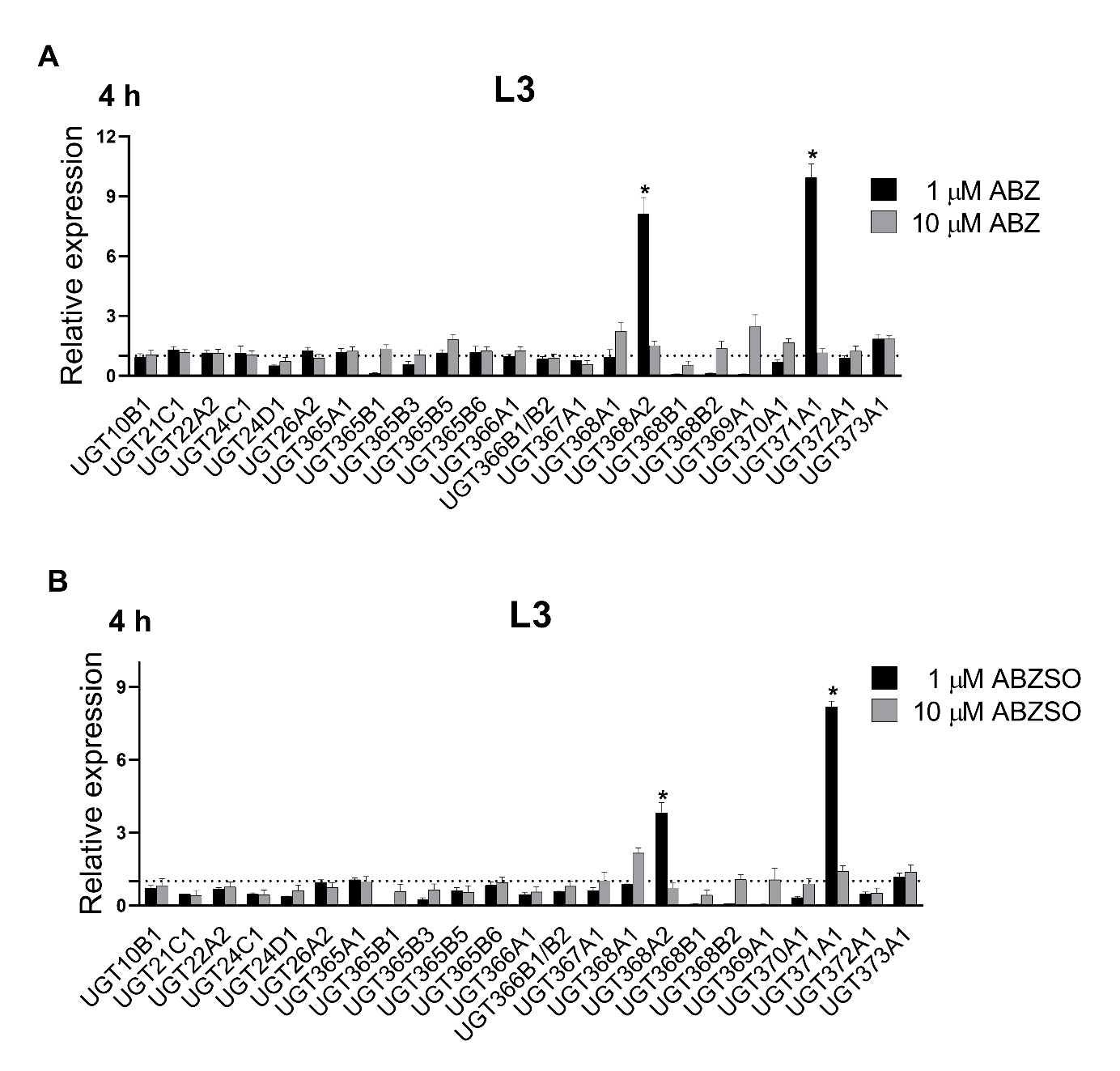


**Supplementary Figure 3.** Changes in the relative amount of the main ABZSO metabolites after 0.5 µM ABZSO incubation of eggs, L1s and L3s of the susceptible ISE and resistant IRE strains. M6_ABZ_ (ABZSO2) (A) and ABZ (B) in nematode’s homogenates and medium. The data represent the mean ± S.E.M (N=3). IS=internal standard, n.d.=not detected, * indicates a significant difference between ISE and IRE, P < 0.05.


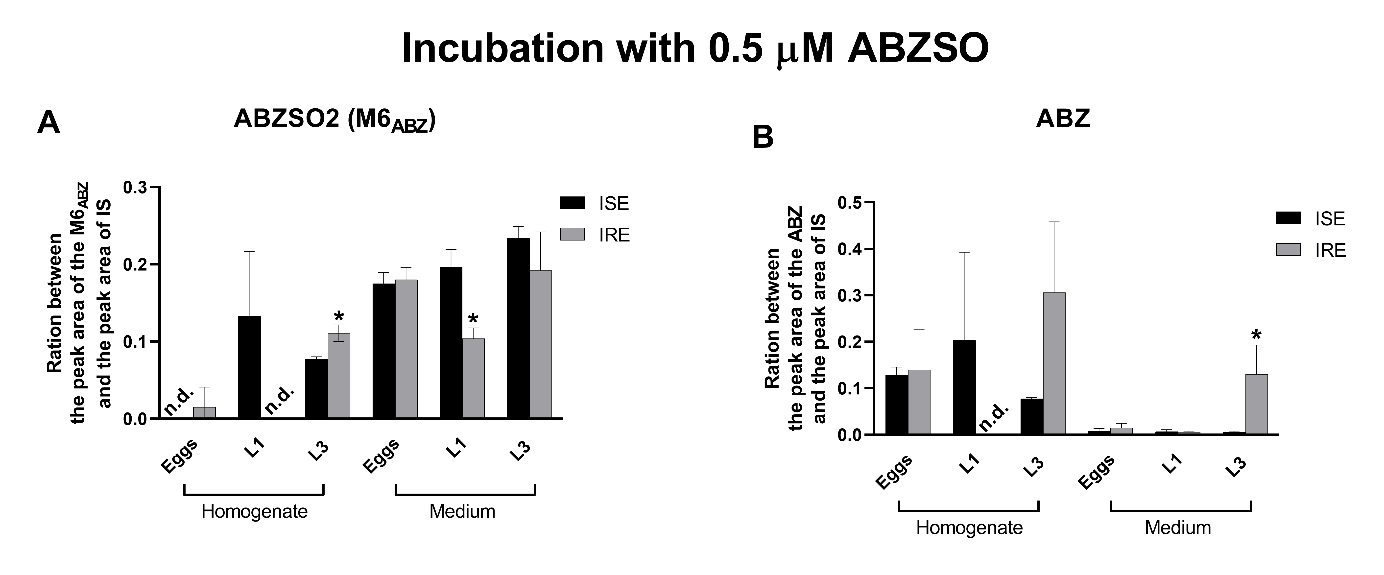


**Supplementary figure 4.** The comparison of the relative amount of the ABZSO metabolite M6_ABZ_ (ABZSO2) in homogenates (A) and medium (B) of the L3s susceptible ISE strain and resistant IRE strain incubated in three different concentration of ABZSO (0.5 µM, 1 µM and 10 µM). The data represent the mean ± S.E.M. (N=3). IS=internal standard, n.d.=not detected, * indicates significant difference between different concentration, # indicates significant differences between ISE and IRE, P < 0.05.

*
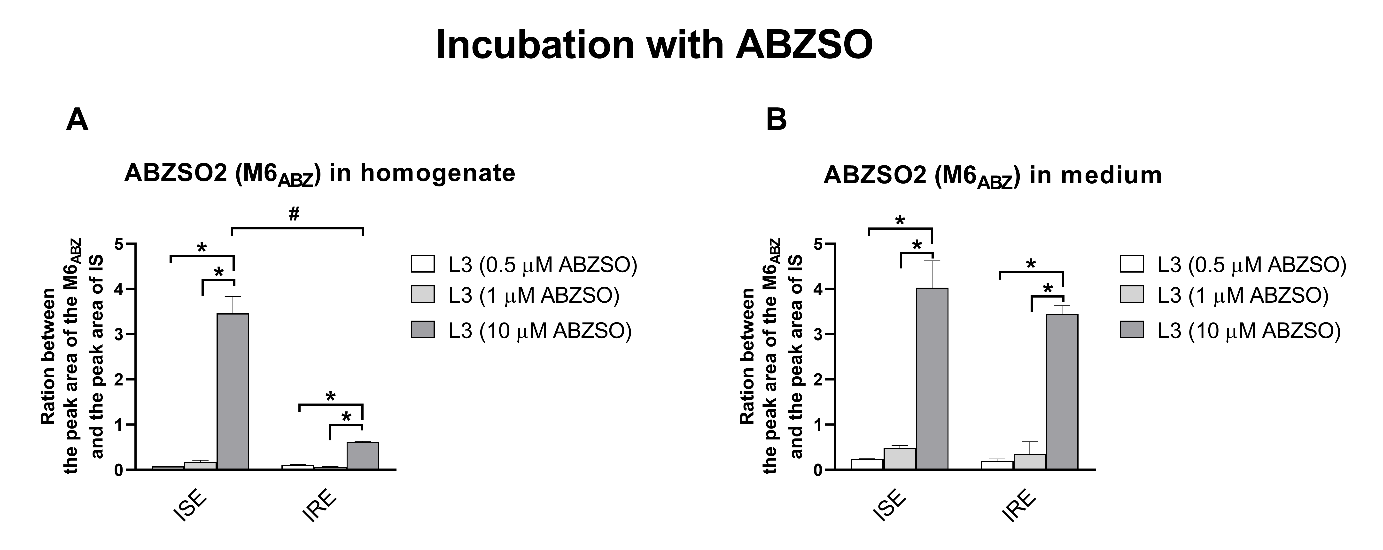
*

**Supplementary figure 5.** The comparison of expression levels of selected UGTs based on the relative quantification by qPCR (left axis, circles and dotted line) and relative quantification based on TPM values (transcript per million -right axes, squares and full line) by differential RNA sequencing (Laing R., et al 2013, Genome Biol. 14:R88).


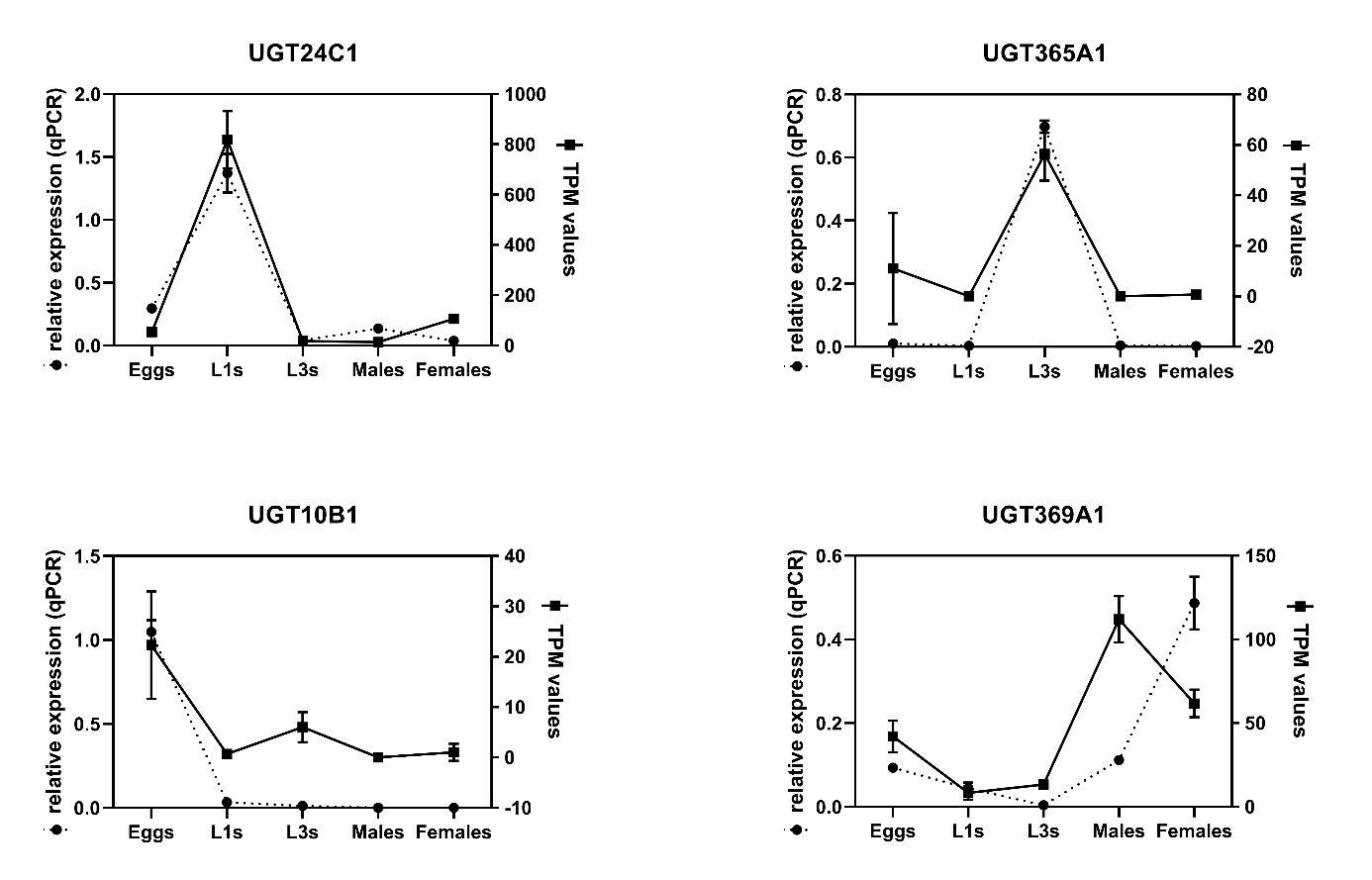


**
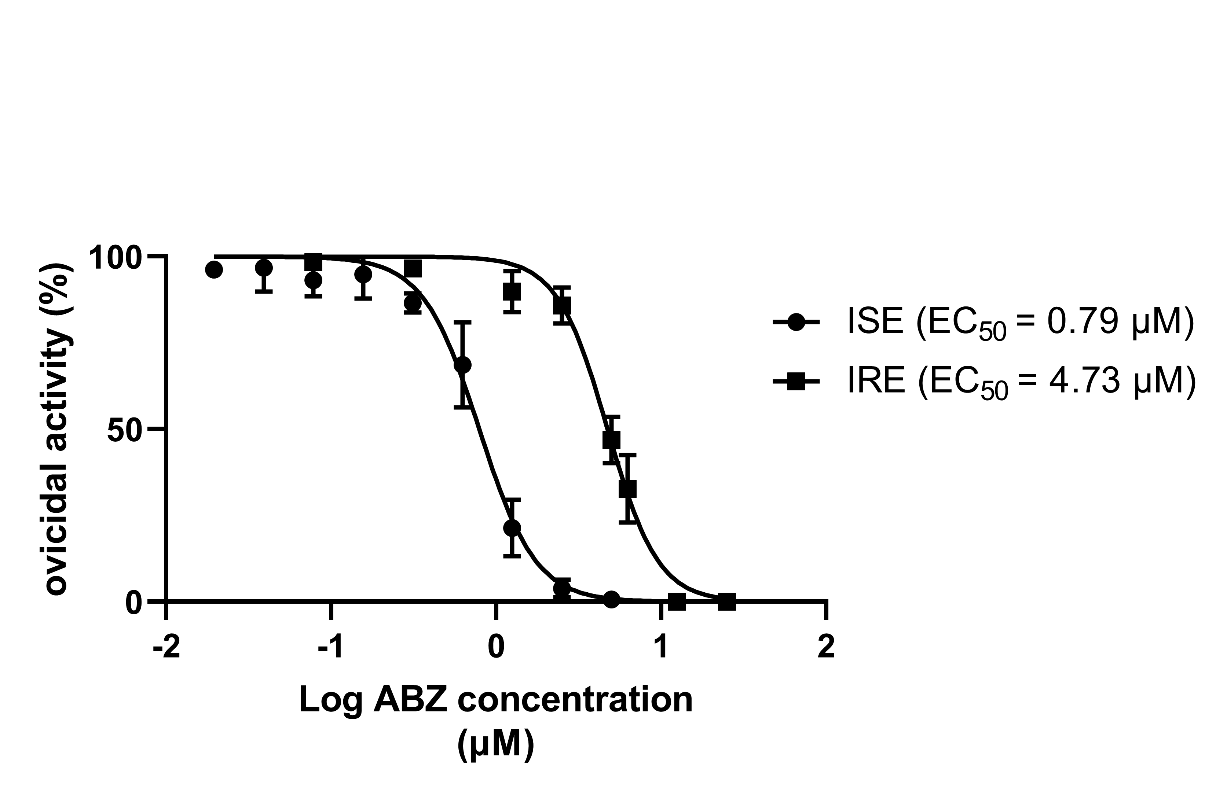
**

**Supplementary figure 6.** Dose-response curves and EC_50_ values obtained for albendazole (ABZ) in ISE and IRE strains.

**Supplementary figure 7.** UHPLC-MS analysis - total ion chromatograms (TIC) of metabolites joint from MRM modes.

**A:** ISE Eggs


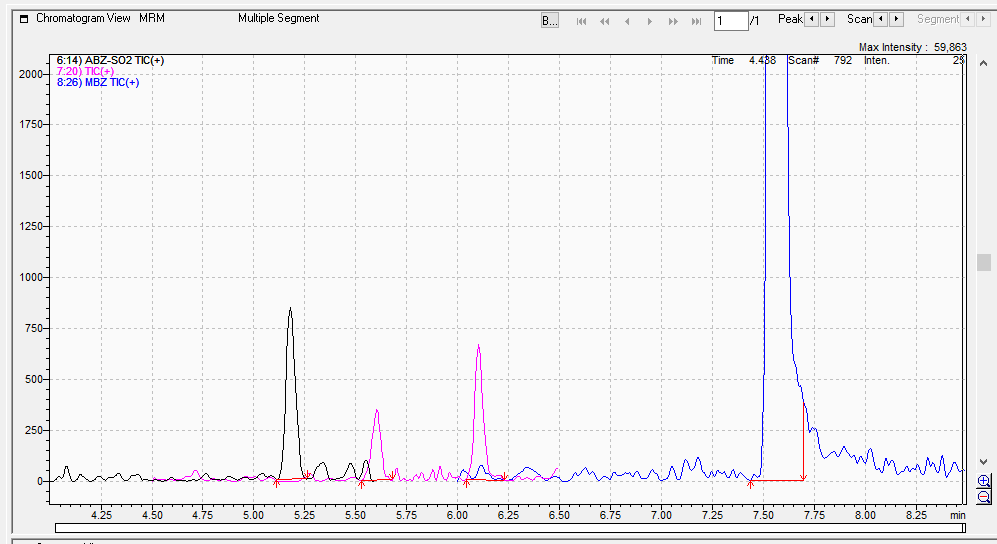


M8_ABZZ_

↘

↘→

M7_ABZZ_

↘

↘→

M6_ABZZ_

↘

↘→

MBZ

**B**: ISE Eggs


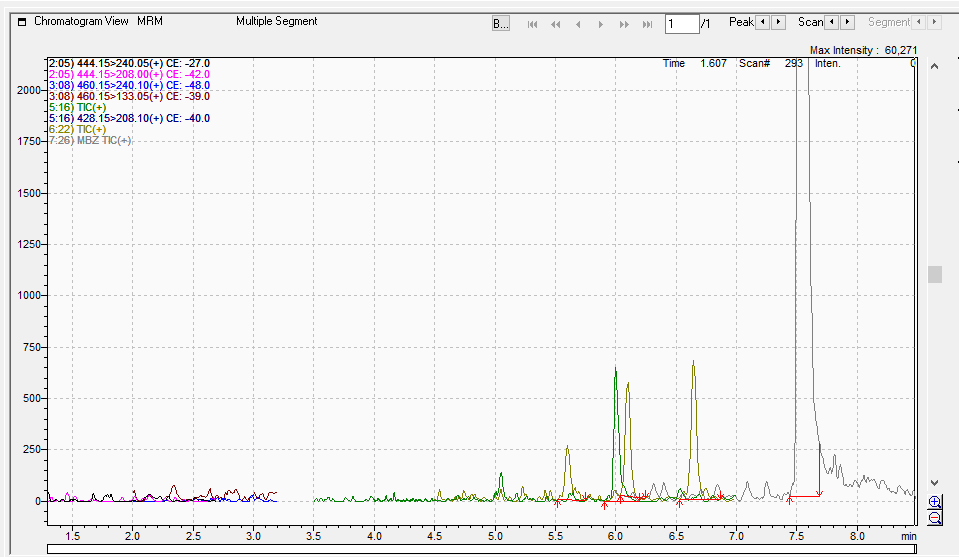


M7_ABZZ_

↘

↘→

M9_ABZZ_

↘

↘→

M8_ABZZ_

↘

↘→

MBZ

M10_ABZ_

↘

↘→

**C**: ISE Eggs


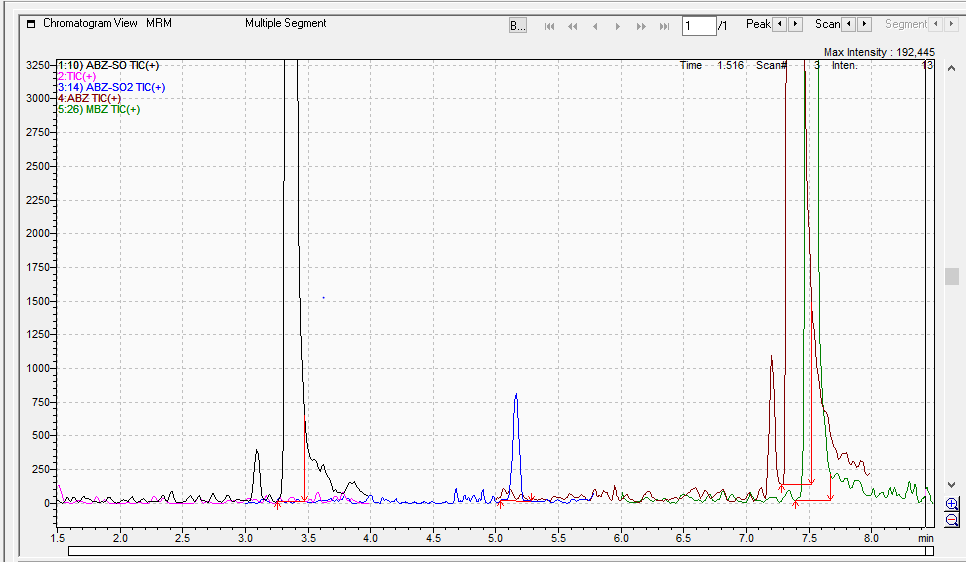


M6_ABZZ_

↘

↘→

MBZ

↘

↘→

ABZ

↘

↘→

M3_ABZZ_

↘

↘→

**D**: IRE Eggs

MBZ

↘

↘→


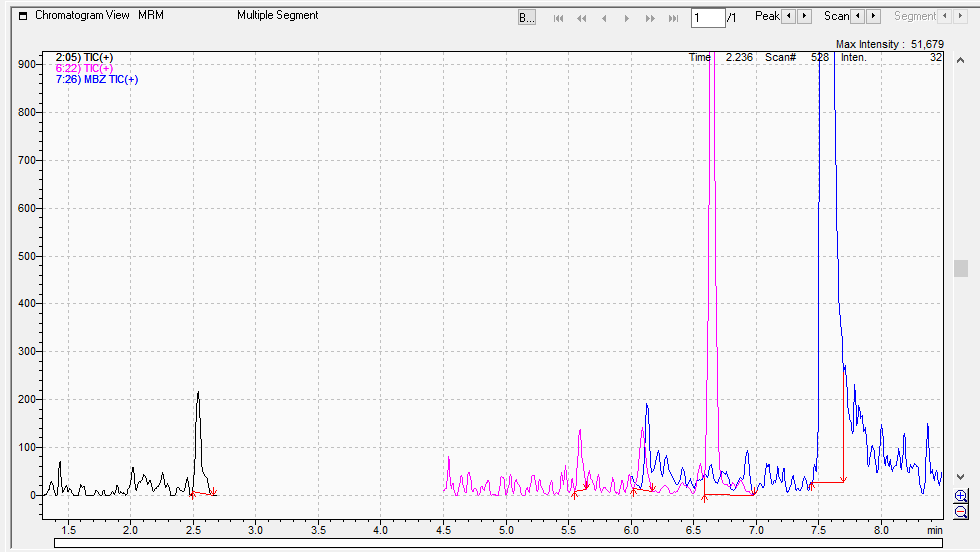


M9_ABZZ_

↘

↘→

M8_ABZZ_

↘

↘→

M7_ABZZ_

↘

↘→

M2_ABZZ_

↘

↘→

| **Supplementary Table 1.** UDP-glycosyltransferases - Gene names and primers | | | | | | |
| --- | --- | --- | --- | --- | --- | --- |
| **Gene ID** | **WORM BASE (**[**PRJEB506)**](https://parasite.wormbase.org/Haemonchus_contortus_prjeb506/Gene/Summary?g=HCOI01574200) | | **Primer Sequence** | **Product size** | **Amplification eff. [%]** | **Source** |
|  | **Current** | **Former** |  |  |  |  |
| UGT10B1 | HCON_00104220 | HCOI01037200 | F: TCAACTTGCAATCACCCACG  R: CTGAGAGCGGGATGAGGTAG | 147 | 104 | Own design |
| UGT21C1 | HCON_00119360 | HCOI01817600 | F: GCCGTGCTGCTGAATTGAAT  R: CGTCTGGATTATCACGAAGAGAG | 69 | 97 | Own design |
| UGT22A2 | HCON_00135760 | HCOI00058000 | F: GATCTCTGTGGAATCGGCGT  R: TATGAAGGTGGTGCTGGGAC | 119 | 100 | Own design |
| UGT24B1 | HCON_00126565 | HCOI00244800 | F: ACGTACACCTATTCCAATGGCT  R: CCTTGGTTTTGGGGTGTTGAAG | 60 | 106 | Own design |
| UGT24C1 | HCON_00122990 | HCOI00868700 | F: GGATCCGAGACATAGGCAGC  R: ACAGTGAGTTTGCATCCCTC | 69 | 93 | Own design |
| UGT24D1 | HCON_00126560 | HCOI01592800 | F: GAGTAGCAGAGACGTTGGCA  R: TCCCGAAATCATCACCATCGT | 59 | 100 | Own design |
| UGT24D2 | HCON_00127680 | HCOI00244900 | F: GATCAGTCGCTCCCAGTCAC  R: TGGGTGGCAATCGATCTTTCA | 132 | 90 | Own design |
| UGT26A2 | HCON_00092310 | HCOI01238200 | F: GGGCCTGGTTGTCTTCTCTC  R: TGTTTCAGTGGGATCGCCTC | 156 | 99 | Own design |
| UGT27B1 | HCON_00132780 | HCOI01933800 | F: TGTGATGGTTTCGGAATGGA  R: AGCACGGTTTTCTCATCAAGA | 60 | 100 | Own design |
| UGT365A1 | HCON_00125590 | HCOI01462600 | F: GCTAGCTACGTTCCAGGAGC  R: CAGACGCTCAAAGGTGTTCA | 63 | 92 | Own design |
| UGT365B1 | HCON_00125530 | HCOI01462400 | F: GCTTGCCAAACACGGTACTG  R: GAATATCCTTCTCAGAGAGTCCC | 139 | 88 | Own design |
| UGT365B2 | HCON_00125570 | HCOI01461700 | F: ACGACGATGAGCTAATGAAACA  R: ACCGCTGAGAATACACCAATTG | 133 | 100 | Own design |
| UGT365B3 | HCON_00125540 | HCOI01461900 | F: TCATTCGGTTCAGCAATCAAGG  R: CGAAGACTTCCAGAAAATTCCTCT | 70 | 100 | Own design |
| UGT365B4 | HCON_00125545 | HCOI01462000 | F: TTCACAAAACCATCCCGCTC  R: CGGCATGTAGATTGATTTAGCCA | 149 | 100 | Own design |
| UGT365B5 | HCON_00125560 | HCOI01462200 | F: CTTGGTGGAATCGCGGTCTA  R: TTTTGAGGTGCCCATGTGGT | 269 | 100 | Own design |
| UGT365B6 | HCON_00125550 | HCOI01462100 | F: TCCAAGTTATGTTCCAGGGCAT  R: AGTTCTTCATAATCCTTGAACCGC | 177 | 100 | Own design |
| UGT366A1 | HCON_00121110 | HCOI02015300 | F: GCAGCCTTTGATGAGCACAC  R: GGCCCACTTGAATGAGACGA | 68 | 100 | Own design |
| UGT366B1/B2 | HCON_00121182 | HCOI00320100 | F: GGCGATCAAATAAGGAATGCG  R: ATCTGGGATTGTTGAGAACTAGG | 127 | 100 | Own design |
| UGT366C1 | HCON_00121250 | HCOI01255100 | F: TTATGCCAGCCTCTCTCGGT  R: CACTTGCGATGCCTGTCTG | 111 | 100 | Own design |
| UGT366D1 | HCON_00121240 | HCOI01255000 | F: CGGGAAGCCATTGATCGAGT  R: TGAAAGGACGTCGCGCTAAT | 88 | 100 | Own design |
| UGT367A1 | HCON_00165360 | HCOI00538800 | F: AGCGAACTCTGGCCTTCATC  R: TCCGCGAACAATGGTATGGT | 97 | 100 | Own design |
| UGT368A1 | HCON_00040966 | HCOI01078900 | F: GGAGTTCCCACCAATCCCAG  R: CGTTCACGTTCAGGAGGACA | 229 | 100 | Own design |
| UGT368A2 | HCON_00040964 | HCOI01452100 | F: TCGGCCAACAGCAAACATTG  R: GTATTGGGGAACGCGGAGAA | 210 | 100 | Own design |
| UGT368B1 | HCON_00040962 | HCOI00785300 | F: TGGGAAGAAAAATTGAGGCAGTT  R: TGTTTCAGCAGCTTCCTTAAATCA | 74 | 100 | Own design |
| UGT368B2 | HCON_00040960 | HCOI00785400 | F: CAGTGGGAAAAGGATCTATGGA  R: TGTTTGAGCAATTCCCGCAG | 77 | 100 | Own design |
| UGT369A1 | HCON_00194230 | HCOI00240800 | F: TCTAGTTTCGTTCCGGCCAC  R: TCGCCCAAGGAACGTCATT | 60 | 100 | Own design |
| UGT370A1 | HCON_00133660 | HCOI01632300 | F: CCACCGAGAATCTGACAGCC  R: GGGCTGCATTTGAGGTGAATC | 69 | 100 | Own design |
| UGT370B1 | HCON_00133650 | HCOI01632400 | F: GAGAAACGGATGGATGGCGA  R: ACGGGCTGCATTCGAGTTAA | 133 | 100 | Own design |
| UGT371A1 | HCON_00108700 | HCOI01917400 | F: CCACACACTCAGCATATCACT  R: GTTCCCTTCGATGTTGGATCA | 160 | 100 | Own design |
| UGT372A1 | HCON_00161690 | HCOI01985800 | F: CTCGTCGTTTGGGTATCGCT  R: CGAAGCTGGTGTCCGTAAGT | 99 | 100 | Own design |
| UGT373A1 | HCON_00040490 | HCOI01651100 | F: TCAACCCTCGGAATGATGCC  R: AATGGTGTCAGTCCGGTTGG | 86 | 100 | Own design |
| *gpd* | CDJ92718.1^*^ |  | F: ACGAGACCTACAATGCAGCC  R: GCGAGACAGTTGGTGGTACA | 67 | 101 | Lecová et al. (2015) |
| *ama* | CDJ91461.1^*^ |  | F: TATGGGAGGTCGTGAAGGTC  R: GTGGGCTTCATAGTGGGCATA | 214 | 104 | Lecová et al. (2015) |
| *ncbp* | CDJ82645.1^*^ |  | F: CCGAGCAGATACCGAAAATGC  R: CGAAGCCTGCATCATAGTCCA | 89 | 99 | Lecová et al. (2015) |

^*^ NCBI GenBank database

**Supplementary table 2**. Biotransformation of ABZSO in *H. contortus* juvenile stages - the metabolites detected by UHPLC-MS/MS.

| **Metabolite designation** | **t_R_ [min]** | **Theoretical *m/z* values of [M+H]^+^ ions** | **Elemental composition** | **Description of metabolite formation** | | **Product ions of [M+H]+, *m/z*** | **Metabolite** |
| --- | --- | --- | --- | --- | --- | --- | --- |
|  |  |  |  | **Phase I** | **Phase II** |  |  |
|  |  |  |  |  |  |  |  |
| M6_ABZ_ | 5.2 | 298.09 | C_12_H_15_N_3_O_4_S | S-oxidation | - | 266, 224, 159 | ABZSO2 |
| ABZ | 7.4 | 266.10 | C_12_H_15_N_3_O_2_S | Reduction of sulfoxide (-O) | - | 234 | ABZ |
| ABZSO (parent drug) | 3.4 | 282.09 | C_12_H_15_N_3_O_3_S | - | - | 240, 208, 191,159 |  |

**Supplementary table 3.** Presence (+) or absence (−) of ABZSO(RCB) metabolites in homogenates and medium of *H. contortus* juvenile stages from ISE and IRE strains.

| **Metabolite designation** |  | | **Homogenate of *H. contortus*** | | | | | | | | | |  | | **Medium** | | | | | | | | | |
| --- | --- | --- | --- | --- | --- | --- | --- | --- | --- | --- | --- | --- | --- | --- | --- | --- | --- | --- | --- | --- | --- | --- | --- | --- |
|  | **ISE** | | | | | |  | | **IRE** | | | | **ISE** | | | | | |  | | **IRE** | | | |
|  | **Eggs**  **(0.5µM)** | **L1/L2**  **(0.5µM)** | | **L3 (0.5µM)** | **L3 (1µM)** | **L3 (10µM)** | **Eggs**  **(0.5µM)** | **L1/L2**  **(0.5µM)** | | **L3 (0.5µM)** | **L3 (1µM)** | **L3 (10µM)** | **Eggs**  **(0.5µM)** | **L1/L2**  **(0.5µM)** | | **L3 (0.5µM)** | **L3 (1µM)** | **L3 (10µM)** | **Eggs**  **(0.5µM)** | **L1/L2**  **(0.5µM)** | | **L3 (0.5µM)** | **L3 (1µM)** | **L3 (10µM)** |
| M6_ABZ_ | - | + | | + | + | + | - | - | | + | + | + | + | + | | + | + | + | + | + | | + | + | + |
| ABZ | + | + | | + | ­+ | + | + | + | | + | + | + | + | + | | + | + | + | + | + | | + | + | + |
| ABZSO | + | + | | + | + | + | + | + | | + | + | + | + | + | | + | + | + | + | + | | + | + | + |
